# Genome Sequences of the *Arthrobacter globiformis* Phage BillyTP (Cluster AY) and *Gordonia rubripertincta* Phage MAnor (Cluster CT)

**DOI:** 10.17912/micropub.biology.001486

**Published:** 2025-03-04

**Authors:** James Melton, Camilla Augustus, Jordan Dotson, Adja Camara, Laila Christian, Damera McClendon, McKenzie Severson, Morgan Wills, Deana Burris, Destinee Williams, Vashti Williams, Shrijeeta Ganguly

**Affiliations:** 1 Biology Department, Spelman College

## Abstract

Phages BillyTP (Cluster AY) and MAnor (Cluster CT) were isolated from soil using *Arthrobacter globiformis* B-2979 and *Gordonia rubripertincta* NRRL B-16540, respectively, as hosts. The genome of BillyTP is 53,003 base pairs (bp) and contains 96 putative genes, while the genome of MAnor is 48,333 bp encoding 73 putative genes. BillyTP and MAnor are assigned to actinobacteriophage clusters AY and CT, respectively, based on gene content similarity (GCS) of at least 35%.

**Figure 1. Plaque and virion morphologies:**
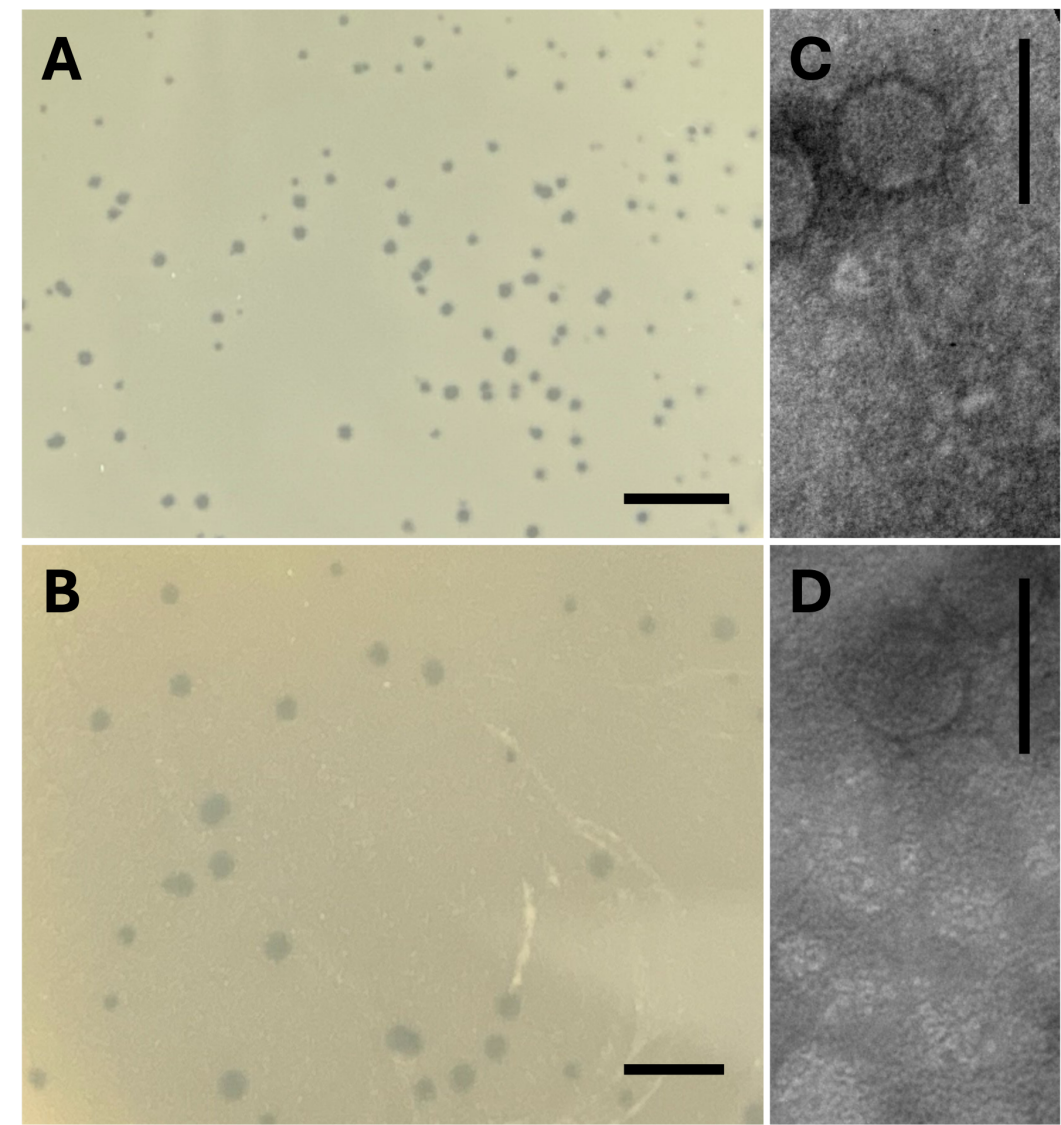
Plaque morphologies (A, B) and electron micrographs (C, D) of phages BillyTP (A, C) and MAnor (B, D). The plaques of BillyTP ranged from 0.5 to 1.5 mm (n=20; average=1 mm; SD=0.28 mm) in diameter, while the plaques of MAnor ranged from 1 to 2.5 mm (n=20; average=2 mm; SD=0.54 mm) in diameter. Virion imaging was performed on a JEOL 100CX II transmission electron microscope (TEM) 100kV and revealed that both phages displayed a typical siphovirus morphology. Scale bars in the plaque images and electron micrographs are 10 mm and 100 nm, respectively. &nbsp; &nbsp;

## Description

*Arthrobacter* and *Gordonia* species (Actinobacteria) are Gram-positive bacteria that are common in soil, and a few species are known to cause rare infections in immunocompromised individuals (Busse et al., 2015; Li et al., 2021; Ramanan et al., 2013) . With antimicrobial resistance (AMR) on the rise, phage discovery is imperative for potential uses to combat infections with phage therapy (Hatfull et al., 2022; Strathdee et al., 2023) . To date, a total of 517 *Arthrobacter* phages and 799 *Gordonia* phages have been isolated and sequenced (Russell and Hatfull, 2017) .

Here, we isolated from soil, imaged, and sequenced phages BillyTP (GPS: 33.655316 N, 84.738776 W) and MAnor (GPS: 33.429512 N, 86.681819 W) using *Arthrobacter globiformis* B-2979 and *Gordonia rubripertincta* NRRL B-16540, respectively, as hosts ( Figure 1 ). Phages were extracted from the soil (0.2 μm filter) after adding peptone-yeast extract-calcium (PYCa) liquid medium and vigorously shaking for one hour at 250 rpm. After adding host bacteria and shaking at 30 ˚C for 48 hours, the enriched cultures were refiltered (0.2 μm filter), and the filtrate was plated in top agar with host bacteria and incubated at 30°C for 2-4 days. Both phages were purified through three rounds of plating. BillyTP displayed plaques that ranged from 0.5 to 1.5 mm in diameter (n=20; average=1 mm; SD=0.28 mm), with plaques on the lower end of the size range appearing turbid while the larger plaques appearing clear ( Figure 1A ). MAnor had clear plaques that ranged from 1 to 2.5 mm in diameter (n=20; average=2 mm; SD=0.54 mm; Figure 1B ). Transmission electron microscopy (TEM) was performed using UranylLess EM stain and showed that both phages had a siphovirus morphology with long, non-contractile tails ( Figure 1C, D ).

DNA was extracted from a high titer lysate (BillyTP: 3.3 x 10 ^10^ PFU/mL; MAnor: 5 x 10 ^10^ PFU/mL) using the Promega Wizard DNA Clean-up kit, prepared for sequencing using the NEB Ultra II FS kit and sequenced on an Illumina platform with v3 reagents, resulting in 364,128 and 295,232 150-base single-end reads for BillyTP and MAnor, respectively. Genome assembly was performed using Newbler v2.9 (Miller et al., 2010) , and the genomic termini were identified using Consed v29 (Gordon and Green, 2013) following Russell (2018). The genome of BillyTP was 53,003 bp (906X coverage; 62.8 GC%) and exhibited a 3’ single-stranded overhang that was nine bases in length (5'-CGCCGGTGA-3'). The genome of MAnor was 48,333 bp (865X coverage; 60.7 GC%) and contained a 3’ single-stranded overhang that was 13 bases in length (5'-CGGCGGTAGGCTT-3').

Genome annotations were performed using DNA Master v5.23.6 (Pope and Jacobs-Sera, 2018) and PECAAN v20240320 (Rinehart et al., 2016) . Open reading frames (ORFs) were determined using GeneMark v3.25 (Besemer and Borodovsky, 2005) and Glimmer v3.02 (Delcher et al., 2007) . Starterator v1.2 was used to compare the starting position in previously annotated genomes. Gene functions were assigned based on BLAST (Altschul et al., 1990) , using the Actinobacteriophage and NCBI non-redundant database, HHPred (Söding et al., 2005) , using the PDB_mmCIF70, Pfam-v.36, NCBI Conserved Domains databases, and Phamerator (Cresawn et al., 2011) , using Actino_draft database v578. Based on gene content similarity (GCS) of at least 35% to phages in the Actinobacteriophage database, phagesDB, BillyTP and MAnor were assigned to clusters AY and CT, respectively (Russell and Hatfull, 2017; Pope et al., 2017) . Default settings were used for all software.

A total of 96 protein-coding genes were predicted in the genome of BillyTP, and 45 genes were assigned a putative function. Of note are two putative tyrosine integrases. An immunity repressor could not be identified; however, two genes encoding helix-turn-helix DNA binding domain-containing proteins are located near the two tyrosine integrase genes, which is similar to most cluster AY phages (Gavin et al., 2024) . Therefore, BillyTP is likely capable of establishing lysogeny. As with a majority of cluster AY phages, the ATPase and nuclease domains of the large terminase are encoded by two separate and consecutive genes. MAnor contains 73 putative protein-coding genes, and 35 genes were assigned a putative function. No immunity repressor or integrase could be identified, suggesting that MAnor is unlikely to establish lysogeny. Consistent with other CT phages, MAnor contains two consecutive lysinA genes, one encoding lysinA with a peptidase domain and the other with a hydrolase domain.

**Nucleotide sequence accession numbers**

BillyTP is available at GenBank with Accession No. PP978841 and Sequence Read Archive (SRA) No. SRR30907830. MAnor is available at GenBank with Accession No. PQ184784 and Sequence Read Archive (SRA) No. SRR30907825.
